# Utility of Serum Vascular Endothelial Growth Factor and Its Clinico-Histopathological Correlation With Cervical Cancer: A Tertiary Care Centre Study

**DOI:** 10.7759/cureus.113209

**Published:** 2026-07-23

**Authors:** Shashikant, Ram Raghuvanshi, Mustafa Ali, Hukam Meena, Radhika Rai, Pankaj Shinde

**Affiliations:** 1 Pathology, Virendra Kumar Sakhlecha Government Medical College, Neemuch, IND; 2 Pathology, Dr. Laxminarayan Pandey Government Medical College, Ratlam, IND; 3 Pathology, Mahatma Gandhi Memorial Medical College, Indore, IND

**Keywords:** biomarker, cervical cancer, enzyme-linked immunosorbent assay (elisa), histopathological parameters, vascular endothelial growth factor

## Abstract

Objective

The present study was conducted to evaluate serum vascular endothelial growth factor (VEGF) as a potential biomarker for cervical cancer and to assess its role as a prognostic factor by correlating serum VEGF levels with other clinicohistopathological parameters in patients with cervical cancer.

Materials and methods

Newly diagnosed patients with cervical cancer confirmed by histopathology in the Department of Pathology over a 12-month period were included in the study. Patients who had not undergone any prior treatment or surgery were enrolled. Serum samples were collected for VEGF estimation, and the analysis was performed using the enzyme-linked immunosorbent assay (ELISA) technique in the Department of Pathology at Mahatma Gandhi Memorial Medical College and Maharaja Yeshwant Rao Holkar Hospital, Indore, India.

Results

A total of 50 patients were included during the study period. VEGF levels were higher than normal in patients with cervical cancer, with a mean value of 670.89 pg/mL. The VEGF levels increased significantly with tumor grade. However, no significant correlation was observed between VEGF levels and the histopathological type of the tumor.

Conclusion

The findings support the potential of serum VEGF as a non-invasive prognostic biomarker that may aid in stratifying patients according to disease severity and guiding individualized management strategies. Its significant correlation with tumor burden suggests that it could serve as a valuable tool for monitoring therapeutic response and disease progression. However, further large-scale, prospective, and longitudinal studies are required to validate its clinical utility and establish its role in routine practice.

## Introduction

According to data from the National Centre for Disease Informatics and Research (NCDIR)-National Cancer Registry Programme (NCRP), the estimated numbers of cervical cancer cases and related deaths in India in 2025 were 79,239 and 42,789, respectively [1]. Globally, cervical cancer ranked eighth in incidence, with 662,301 cases, and ninth in mortality, with 348,874 deaths. Asia reported the highest incidence and mortality rates compared with other continents [2]. The cervix is the lower part of the uterus that connects the uterus to the vagina and is divided into the ectocervix and endocervix, which are lined by squamous and columnar epithelium, respectively. Between these regions lies the transformation zone (squamocolumnar junction), which is the primary site of cervical carcinogenesis [3]. Certain types of human papillomavirus (HPV) are responsible for more than 99% of cervical cancer cases. HPV is primarily transmitted through sexual contact, and transmission can occur even without penetrative intercourse, as skin-to-skin contact alone may be sufficient [4,5]. Vascular endothelial growth factor (VEGF) is one of several angiogenic factors that play an essential role in tumor angiogenesis [6]. Tumor angiogenesis is a critical process that influences both the malignant potential of gynecological cancers and patient survival [7].

## Materials and methods

This study was conducted in the Department of Pathology at Mahatma Gandhi Memorial Medical College and Maharaja Yeshwant Rao Holkar Hospital, Indore, India, over a period of 12 months from 1^st^ December 2023 to 30^th^ November 2024, following approval from the Institutional Scientific and Ethics Committee (EC/MGM/Nov-2023/218).

The sample size was calculated using Hulley's formula for a correlation coefficient $N = \left( \frac{Z_{\alpha} + Z_{1-\beta}}{C} \right)^{2} + 3$, where, N is the required sample size; Zα is the standard normal deviation corresponding to the chosen level of significance (1.96 for a two-sided α = 0.05); Z1−β is the standard normal deviation corresponding to the desired study power (0.84 for 80% power); $\mathrm{C} = 0.5 \times \ln \left( \frac{1 + \mathrm{r}}{1 - \mathrm{r}} \right)$, where r is the anticipated correlation coefficient used for sample size estimation. This yielded a minimum required sample size of 50 participants. 

Inclusion criteria

Patients aged ≥18 years with histologically confirmed cervical cancer on biopsy specimen in the Department of Pathology, Mahatma Gandhi Memorial Medical College, and Maharaja Yeshwant Rao Holkar Hospital over a period of 12 months, who provided informed consent, were enrolled in the study.

Exclusion criteria

Patients with a negative or inconclusive cervical biopsy report, a history of prior surgical treatment, chemotherapy, or radiotherapy, or those unwilling to provide informed consent were excluded from the study.

After recording the relevant demographic, clinical, and physical data, serum samples were collected from the median cubital veins in the antecubital fossa using yellow-topped clot activator serum separator tubes and immediately centrifuged at 3000 rpm for 10 min at 4°C. The aliquoted supernatant (serum) was stored at −80°C until further analysis using an enzyme-linked immunosorbent assay (ELISA). Serum VEGF levels were measured using a Human VEGF ELISA Kit (Catalogue No. EH0327, Revision V4.0, 48T/96T; FineTest, Wuhan Fine Biotech Co., Ltd., Wuhan, Hubei, China) according to the manufacturer's instructions. The tumors were histologically classified into two types: squamous cell carcinoma (SCC) and adenocarcinoma. Histopathological grading (Grades I-III) was performed by a group of pathologists based on the degree of keratinization in squamous cell carcinoma and the extent of glandular differentiation in adenocarcinoma, while blinded to the serum VEGF values (Table 1).

**Table 1:** Criteria for histopathological grading of squamous cell carcinoma and adenocarcinoma

Grade	Squamous cell carcinoma	Adenocarcinoma
Grade I	Keratinization in >50% of the tumor	Glandular differentiation in >50% of the tumor
Grade II	Keratinization in 10%-50% of the tumor	Glandular differentiation in 10%-50% of the tumor
Grade III	Keratinization <10% of the tumor	Glandular differentiation in <10% of the tumor

The collected data were entered into Microsoft Excel (Microsoft Corporation, Redmond, WA, USA) and analyzed using IBM SPSS Statistics software, version 23.0 (IBM Corporation, Armonk, NY, USA). Continuous variables were summarized as mean ± standard deviation (SD). Analysis of Variance (ANOVA) was employed to compare the means across multiple groups. Pearson's correlation analysis was conducted to evaluate the association between serum VEGF levels and clinico-histopathological parameters in cervical cancer. A p-value of less than 0.05 was considered statistically significant.

## Results

The majority of the study participants belonged to the 51-75 years age group, accounting for 58% of the total cases. This was followed by the 31-50 years age group, which comprised 38% of the participants. Only 2% of the cases were observed in each of the 18-30 years and >76 years age groups (Table 2).

**Table 2:** Age-wise distribution of the study participants (n = 50)

Age Group	Number (n)	Percentage (%)
18-30 years	1	2.0
31-50 years	19	38.0
51-75 years	29	58.0
>76 years	1	2.0
Total	50	100.0

In the present study, the majority of the participants (66%) were postmenopausal, while 34% were premenopausal. This may suggest a possible association between menopausal status and the occurrence of cervical cancer (Table 3).

**Table 3:** Frequency of the study participants based on menopausal status

Menopausal status	Number (n)	Percentage (%)
Postmenopausal	33	66.0
Premenopausal	17	34.0
Total	50	100.0

In this study, 56% of cervical cancer cases were classified as Grade 2 (moderately differentiated), making it the most common histological grade. Grade 3 (poorly differentiated) tumors accounted for 24% of cases, while Grade 1 (well-differentiated) tumors were observed in 20% of participants. These findings suggest that moderately differentiated tumors predominated in the study population (Table 4).

**Table 4:** Histopathological grade distribution among the study participants (n=50)

Histopathological Grading	Number (n)	Percentage (%)
Grade 1 (Well-differentiated)	10	20.0
Grade 2 (Moderately differentiated)	28	56.0
Grade 3 (Poorly differentiated)	12	24.0
Total	50	100.0

Squamous cell carcinoma was the predominant histological subtype among the study participants, accounting for 88% of the cases, while adenocarcinoma constituted 12% (Table 5).

**Table 5:** Distribution of the study participants based on histological type (n=50)

Histopathological Type	Number (n)	Percentage (%)
Adenocarcinoma	6	12.0
Squamous cell carcinoma	44	88.0
Total	50	100.0

The mean serum VEGF level among the study participants was 670.89 pg/mL, with a standard deviation of 317.12 pg/mL, indicating considerable variability among the patients. Serum VEGF levels ranged from 196.94 pg/mL to 1387.61 pg/mL, with an overall range of 1190.67 pg/mL. These findings suggest substantial variation in serum VEGF levels among cervical cancer patients, which may reflect differences in tumor characteristics, disease stage, and aggressiveness (Table 6).

**Table 6:** Serum vascular endothelial growth factor (VGEF) levels among study participants

VEGF level				
Mean	SD	Minimum	Maximum	Range
670.8912	317.12365	196.94	1387.61	1190.67

The mean serum VEGF levels increased progressively with higher histopathological grades of cervical cancer. Grade 1 (well-differentiated) tumors had the lowest mean serum VEGF level (431.83 pg/mL), followed by Grade 2 (moderately differentiated) tumors (630.19 pg/mL), while Grade 3 (poorly differentiated) tumors exhibited the highest mean level (944.36 pg/mL). The p-value of 0.001 indicates a statistically significant association between serum VEGF levels and tumor grade. These findings suggest that higher serum VEGF levels are associated with higher histopathological grades and may reflect increased tumor aggressiveness in poorly differentiated tumors (Table 7).

**Table 7:** Mean serum vascular endothelial growth factor (VGEF) levels in different histopathological grades

VEGF Level				
Histopathological Grade	Mean	SD	F	(ANOVA) p-value
Grade 1	431.83	236.60	11.82	0.001**
Grade 2	630.19	274.19		
Grade 3	944.36	260.94		
Total	670.89	317.12		

The mean serum VEGF level was higher in patients with adenocarcinoma (785.18 pg/mL) than in those with squamous cell carcinoma (655.31 pg/mL). However, the p-value of 0.352 indicates that the observed difference was not statistically significant. These findings suggest that, although serum VEGF levels were higher in patients with adenocarcinoma, the difference between the two histological types was insufficient to establish a significant association in the study population (Table 8).

**Table 8:** Mean serum vascular endothelial growth factor (VGEF) levels in different histopathological types

VEGF Level				
Histopathological Types	Mean	SD	t	(ANOVA) p-Value
Adenocarcinoma	785.18	388.34	0.94	0.352
Squamous cell carcinoma	655.31	308.21		

## Discussion

Cervical cancer remains one of the most prevalent malignancies affecting women, particularly in low-resource settings where routine screening and early diagnostic facilities are often limited. In the pursuit of improving diagnostic precision and understanding tumor biology, angiogenic markers such as VEGF have emerged as promising tools. VEGF plays a pivotal role in neovascularization by promoting blood vessel formation within the tumor microenvironment, thereby facilitating tumor progression [8].

Recognizing its biological significance, the present study was conducted to evaluate serum VEGF levels in patients with cervical cancer and to investigate their relationship with various clinico-histopathological parameters. By establishing these correlations, the study aims to elucidate the potential of VEGF as a supportive biomarker for disease monitoring and prognostication in a tertiary care setting. In the present study involving 50 participants, the majority (58%) belonged to the 51-75 years age group, with a mean age of 53.78 ± 10.77 years, indicating a higher prevalence of cervical cancer among middle-aged and older women. This finding suggests a possible association between increasing age and the occurrence of cervical cancer. Additionally, 66% of the study participants were postmenopausal, highlighting a higher prevalence of cervical cancer among postmenopausal women. This finding suggests a potential association between menopausal status and the development of cervical cancer. Menopause itself does not cause cervical cancer. However, the combined effects of old age, hormonal changes, and modification in the local vaginal environment may reduce immune scrutiny, thereby increasing the likelihood that a pre-existing HPV infection persists for a prolonged period. Persistent HPV infection is a well-established risk factor for the development and progression of cervical cancer [9]. In the present study, histopathological grading revealed that the majority of cervical cancer cases (56%) were moderately differentiated (Grade 2), followed by poorly differentiated tumors (Grade 3) in 24% of cases and well-differentiated tumors (Grade 1) in 20% of cases. The mean serum VEGF level among the study participants was 670.89 ± 317.12 pg/mL. This value is comparable to the findings of Du et al., who reported a median pretreatment serum VEGF level of 647.15 pg/mL in 37 patients with cervical cancer [10]. A progressive and statistically significant increase in serum VEGF levels was observed with advancing histological grade. Grade 1 tumors exhibited the lowest mean VEGF concentration (431.83 pg/mL), followed by Grade 2 tumors (630.19 pg/mL), while Grade 3 tumors demonstrated the highest mean VEGF level (944.36 pg/mL) (p = 0.001). This significant correlation highlights the potential of VEGF as a biomarker of tumor aggressiveness, suggesting that higher VEGF levels are associated with poorer cellular differentiation and a potentially worse prognosis. Similar trends have been reported in previous studies. Bachtiary et al. demonstrated significantly elevated serum VEGF levels in poorly differentiated tumors (median 438 pg/mL) compared with moderately and well-differentiated tumors (median 210 pg/mL), reinforcing the association between VEGF expression and histological grade (p = 0.03) [11]. Likewise, Goncharuk et al. reported increased VEGF expression in poorly differentiated cervical carcinomas, with strong VEGF positivity observed in up to 85% of poorly differentiated cases [12]. These findings are consistent with those of the present study and further support the role of VEGF in tumor progression and angiogenesis. Though the mean serum VEGF level was higher in patients with adenocarcinoma (785.18 pg/mL) than in those with squamous cell carcinoma (655.31 pg/mL), the difference was not statistically significant. Although numerous studies have investigated VEGF expression in different histological types, only a few have evaluated quantitative serum VEGF levels across various tumor histologic types. Santin et al. reported a highly significant difference in VEGF secretion between fresh adenocarcinomas (mean = 2712 pg/mL) and fresh squamous cell carcinomas (mean = 575 pg/mL) [13].

## Conclusions

This study highlights the clinical relevance of serum VEGF as a potential biomarker in cervical cancer. Elevated serum VEGF levels were found to be significantly associated with adverse clinicopathological features, including higher histological grade, menopausal status, and advanced age. Although the differences in serum VEGF levels among histological subtypes were not statistically significant, the overall trend suggests that VEGF may serve as a marker of tumor aggressiveness and disease severity. The current study supports the association between serum VEGF levels and histopathological grading in cervical cancer. Elevated VEGF levels in poorly differentiated tumors may reflect increased angiogenic activity and tumor aggressiveness, establishing VEGF not only as a potential prognostic biomarker but also as a candidate for targeted therapeutic intervention in advanced or aggressive cervical cancers. Serum VEGF shows considerable promise as a non-invasive biomarker for assessing disease severity and progression in cervical cancer. To establish its routine clinical utility, further large-scale, longitudinal studies are warranted. This may open new avenues for incorporating VEGF inhibitors as adjunctive therapies in future cervical cancer treatment protocols.

## References

[1] (2026). Measures taken against rising cervical cancer. https://www.pib.gov.in/PressReleasePage.aspx?PRID=2237400&reg=3&lang=1.

[2] (2022). Global Cancer Observatory. Cancer Today Globocon.

[3] Jenkins D (2007). Histopathology and cytopathology of cervical cancer. Dis Markers.

[4] Okunade KS (2020). Human papillomavirus and cervical cancer. J Obstet Gynaecol.

[5] Burd EM (2003). Human papillomavirus and cervical cancer. Clin Microbiol Rev.

[6] Lugano R, Ramachandran M, Dimberg A (2020). Tumor angiogenesis: causes, consequences, challenges and opportunities. Cell Mol Life Sci.

[7] Delli Carpini J, Karam AK, Montgomery L (2010). Vascular endothelial growth factor and its relationship to the prognosis and treatment of breast, ovarian, and cervical cancer. Angiogenesis.

[8] Burmeister CA, Khan SF, Schäfer G, Mbatani N, Adams T, Moodley J, Prince S (2022). Cervical cancer therapies: current challenges and future perspectives. Tumour Virus Res.

[9] Mukhopadhyay S (2019). A demographic and clinico pathological study of HPV associated cofactors in the pathogenesis of cervical cancer. Indian J Obstet Gynecol Res.

[10] Du K, Gong HY, Gong ZM (2014). Influence of serum VEGF levels on therapeutic outcome and diagnosis/prognostic value in patients with cervical cancer. Asian Pac J Cancer Prev.

[11] Bachtiary B, Selzer E, Knocke TH, Pötter R, Obermair A (2002). Serum VEGF levels in patients undergoing primary radiotherapy for cervical cancer: impact on progression-free survival. Cancer Lett.

[12] Goncharuk IV, Vorobjova LI, Lukyanova NY, Chekhun VF (2009). Vascular endothelial growth factor exression in uterine cervical cancer: correlation with clinicopathologic characteristics and survival. Exp Oncol.

[13] Santin AD, Hermonat PL, Ravaggi A, Pecorelli S, Cannon MJ, Parham GP (1999). Secretion of vascular endothelial growth factor in adenocarcinoma and squamous cell carcinoma of the uterine cervix. Obstet Gynecol.

